# Diversity and Relationships of Eggplants from Three Geographically Distant Secondary Centers of Diversity

**DOI:** 10.1371/journal.pone.0041748

**Published:** 2012-07-27

**Authors:** Maria Hurtado, Santiago Vilanova, Mariola Plazas, Pietro Gramazio, H. Hemal Fonseka, Ramya Fonseka, Jaime Prohens

**Affiliations:** 1 Instituto de Conservación y Mejora de la Agrodiversidad Valenciana, Universitat Politècnica de València, Valencia, Spain; 2 Horticultural Crop Research and Development Institute, Gannoruwa, Sri Lanka; 3 Department of Crop Science, Faculty of Agriculture, University of Peradeniya, Peradeniya, Sri Lanka; United States Department of Agriculture, United States of America

## Abstract

Eggplant (*Solanum melongena* L.) was domesticated in the Indo-Birmanian region, which is also the primary center of diversity for this crop. From there eggplant spread to other regions, and diversity accumulated in several secondary centers of diversity. We have assessed the diversity and relationships of 52 accessions of eggplant from three geographically distant secondary centers of diversity (China, Spain, and Sri Lanka) using 28 morphological descriptors and 12 highly polymorphic genomic SSRs. A wide variation was found for most morphological traits, and significant differences among the three centers of diversity were detected for 22 of these traits. The PCA analysis showed that eggplants from the three origins were morphologically differentiated, and accessions from each of the three secondary centers of diversity presented a typical combination of morphological characteristics. In this respect, discriminant analysis showed that accessions could be correctly classified to their origin using only six traits. The SSR characterization identified 110 alleles and allowed obtaining a unique genetic fingerprint for each accession. Many alleles were found to be private to each origin, but no universal alleles were found for any of the origins. The PCA analysis showed that the genetic differentiation among origins was less clear than for morphological traits, although the analysis of the population structure shows that accessions mostly group according to the origin, but also provides evidence of migration among the three secondary centers of diversity. The genetic diversity (H_T_) within each origin was high, ranging between H_T_ = 0.5400 (Sri Lanka) and H_T_ = 0.4943 (China), while the standardized genetic differentiation (G’_ST_) among origins was moderate (G’_ST_ = 0.2657). The correlation between morphological and SSR distances was non-significant (r = 0.044), indicating that both data are complementary for the conservation of germplasm and breeding of eggplant. These results are relevant for the management of genetic resources, breeding programmes, and evolutionary studies of eggplant.

## Introduction

Eggplant (*Solanum melongena* L.) is the most important *Solanum* crop native to the Old World, and it ranks as one of the most important vegetable crops in the world with an annual worldwide production of more than 41·10^6^ t (2010; FAO data). This results in an average production of 6.1 kg/person/year or 16.7 g/person/day. Eggplant is one of the vegetables with highest antioxidant capacity [1], which is a consequence of its high content in phenolics [2]. Despite the economic and nutritional importance of eggplant, breeding efforts in this crop have been limited [3]. The use of exotic germplasm in breeding programmes can be of great relevance for the improvement of the crop and for addressing future breeding challenges. In this respect, Muñoz-Falcón et al. [4] found that the genetic diversity of modern cultivars of black eggplants was reduced, and that incorporation of black fruit materials from different origins could increase the genetic base of this cultivar type and contribute to better exploitation of the heterosis resulting from the crosses of genetically distant materials [5].

Assessment of the diversity and relationships of the cultivated species facilitates the establishment of conservation strategies, the use of genetic resources in breeding programmes, and the study of the crop evolution. The domestication and evolution of eggplant has been the subject of a number of studies in which historical [6], [7], morphological [8], and molecular [9], [10] data have been used. There is general consensus that eggplant was domesticated in South-East Asia from the wild relative *Solanum incanum* L, and morphological [8], and molecular [9]–[14] data, as well as the high fertility of F1 hybrids with *S. melongena* [3], [8] support this hypothesis. It is unknown how *S. incanum*, which is naturally distributed in Africa and the Middle East, reached the Indo-Birmanian region, although it has been speculated that it could have arrived there unintentionally as a weed or dragged by ocean currents from Africa to India, or intentionally because of the value of its berries for tanning hides [8], [15]. In any case, as occurred with tomato [16], the domestication of eggplant outside of the area of natural distribution of its wild ancestor resulted in an important genetic bottleneck [3], [8].

Diffusion of the crop from its primary center of diversity, situated in the Indo-Birmanian region [17], [18], where primitive cultivars and weedy forms exist [3], [8], [10], to other areas resulted in the diversification of the crop due to micro-evolutive forces like mutation, selection (natural and artificial), genetic drift, or gene flow, and to recombination, and led to the accumulation of diversity in several secondary centers of diversity [10], [19]. For example, the cultivation of eggplant in China has been documented since more than 2000 years ago [7]; its introduction into Europe, through Spain, was brought about by the Arabs before the 10^th^ century [19]. The evolution of the crop in geographically distant secondary centers of diversity led to the differentiation of cultivar types specific to different regions of the world [3]. In this respect, two large groups of eggplant cultivars are considered by breeders: “Occidental” or “Western” eggplants (Middle East, Africa, Europe, and America) and “Oriental” or “Asian” eggplants (Eastern and Southeastern Asia) [3], [6], [10]. Within each of these large groups, several cultivar types are distinguished [3].

Although a number of studies have been devoted to the study of the diversity of eggplant materials from specific countries or regions, like India [18], Spain [19], China [20], [21], Turkey [22], or Asia [10], [23], up to now no comprehensive assessment has been performed on the comparative diversity and regional differentiation of eggplant materials from different regions of the world. The study of the diversity of geographically distant centers of diversity, where eggplant was introduced through different routes, can provide information of interest for understanding the structure of variation in eggplant, as well as for the conservation of genetic resources and breeding of this crop. Similar studies have been undertaken in other crops, such as lentil (*Lens culinaris* Med.) [24], coffee (*Coffea arabica* L.) [25], maize (*Zea mays* L.) [26] and sorghum (*Sorghum bicolor* (L.)) [27].

The characterization of eggplant with both morphological descriptors and molecular markers has proved useful for the study of the diversity and relationships of different varietal groups of eggplant, as they sample different levels of diversity [4], [19], [28], [29]. Availability of characterization data for traits of agronomic interest is essential for breeding programs. Morphological descriptors for the characterization of eggplant are available as a result of the European Eggplant Genetic Resources Network (EGGNET) [30]. These descriptors have been used and validated in a number of characterizations of eggplant genetic resources and breeding materials [4], [19], [28], [29], [31]. Also, molecular markers are useful to study the genetic diversity of eggplant [4], [10]–[12], [14], [20]–[23], [28], [29], [32]–[35]. SSRs have revealed as the best presently available markers to study the relationships of different groups of cultivars of eggplant, as they have wide genome coverage, are highly variable, have a highly repeatability, are easy to use, and are amenable to high throughput [36], [37]. For example, Muñoz-Falcón et al. [28], [29], [38] have found that SSRs are much better than AFLPs at resolving the relationships between and within cultivar groups as well as for assigning correctly accessions to their cultivar groups. Also, Demir et al. [22], when comparing SSRs with RAPDs, found that the former were more adequate than the latter to study the diversity and relationships of local landraces. Several hundred SSR markers are available in eggplant [14], [23], [32]–[35], [39], and among them genomic SSRs have proved to be more polymorphic than EST-SSRs [38].

Here we assess the morphological and the molecular (SSR) diversity of a collection of eggplants from three geographically distant secondary centers of diversity: China, Spain, and Sri Lanka. Our objective is to obtain information on the diversity, relationships, and differentiation among accessions of these three different origins. These results will be of interest for the conservation of genetic resources, breeding, and study of the evolution of the cultivated eggplant.

## Results

### Morphological Characterization

The collection of eggplants studied, as well as each of the three groups of accessions originating from China, Spain, or Sri Lanka, displayed a wide variation for most of the morphological traits studied (Table 1). Although for the 28 morphological traits considered there is overlap in the range of variation of individual accessions among the three groups, significant differences among means of the three origins are found for 22 of the morphological traits considered (Table 1). In this respect, the number of significant differences among origin means for the traits studied has been of 12 for China vs. Spain accessions, 20 for China vs. Sri Lanka accessions, and 15 for Spain vs. Sri Lanka accessions.

**Table 1:** Mean and range for the traits measured in the eggplant accessions originating from China, Spain, and Sri Lanka used for the present study, and probability of the *F*-statistic, obtained from ANOVA analyses, for differences among means.

Trait	Code	China		Spain		Sri Lanka		Prob. *F*
		Mean^a^	Range	Mean^a^	Range	Mean^a^	Range	
No. of accessions		20		14		18		
Plant growth habit	P-Habit	2.1a	1.0–4.2	1.6a	0.7–5.0	2.1a	1.0–5.0	0.394
Plant height (cm)	P-Height	81.2a	48.3–108.2	105.4b	45.0–149.5	95.2b	61.2–120.3	0.006
Shoot tips anthocyanins intensity	P-Anthocyans	6.2b	1.0–9.0	5.1ab	3.0–9.0	4.0a	1.0–7.0	0.010
Stem prickles	P-Prickles	2.3b	0.0–3.0	2.6b	0.0–3.0	0.7a	0.0–3.0	<0.001
Leaf pedicel length (cm)	L-Pedicel	4.9a	2.9–6.2	7.2c	5.1–9.7	5.6b	4.3–6.7	<0.001
Leaf blade length (cm)	L-Length	15.9a	11.6–20.2	20.7c	17.6–28.2	19.2b	17.3–21.9	<0.001
Leaf blade breadth (cm)	L-Breadth	9.4a	5.8–13.3	12.2b	9.4–15.1	12.3b	9.8–14.6	<0.001
Leaf blade length/breadth ratio	L-Ratio	1.71b	1.43–2.01	1.70b	1.53–1.88	1.58a	1.35–1.86	0.005
Leaf blade lobing	L-Lobing	3.5a	1.0–7.0	4.9b	3.0–7.0	4.8b	3.0–7.0	0.009
Leaf anthocyanins intensity	L-Anthocyans	6.9c	0.0–9.0	4.6b	0.0–9.0	2.4a	0.0–9.0	<0.001
Flowers per inflorescence	Fl-Number	1.4a	1.0–4.0	2.5b	1.0–6.0	2.8b	1.5–4.0	0.001
Flower diameter (cm)	Fl-Diameter	3.9a	3.0–5.2	4.1a	2.5–5.9	3.5a	2.5–5.7	0.162
Petals per flower	Fl-Petals	5.5a	5.0–7.0	6.1b	5.7–7.0	5.3a	5.0–6.0	<0.001
Corolla colour	Fl-Corolla	3.4a	1.0–5.0	2.9a	0.0–5.0	3.2a	1.0–7.0	0.606
Fruit longitudinal perimeter (cm)	Fr-Perimeter	30.2a	22.9–46.4	26.8a	17.6–38.7	29.7a	20.6–36.4	0.253
Fruit length (cm)	Fr-Length	13.3a	6.9–22.7	12.5a	6.4–18.9	16.2b	7.6–20.7	0.019
Fruit breadth (cm)	Fr-Breadth	7.6b	3.0–13.5	7.6b	5.5–10.0	5.2a	3.5–8.6	0.002
Fruit length/breadth ratio	Fr-Ratio	2.26a	0.58–5.00	1.70a	0.85–2.99	3.42b	0.89–4.73	<0.001
Fruit weight (g)	Fr-Weight	426b	139–982	332ab	61–685	209a	97–392	0.030
Relative fruit calyx length	Fr-CLength	2.6a	1.0–5.0	5.7b	1.0–9.0	3.1a	1.0–9.0	<0.001
Fruit calyx anthocyanins intensity	Fr-CAnthocyans	7.6c	0.0–9.0	3.6b	0.0–9.0	0.6a	0.0–9.0	<0.001
Fruit colour intensity under calyx	Fr-UnderC	0.7a	0.0–5.0	2.1b	0.0–5.0	4.2c	3.0–5.0	<0.001
Fruit skin chlorophyll	Fr-Chlorophyll	4.3b	0.0–9.0	4.4b	0.0–9.0	0.0a	0.0–0.0	<0.001
Fruit skin L*primary colour	Fr-L*	25.8a	19.6–58.5	34.1a	21.3–54.7	52.5b	22.3–74.8	<0.001
Fruit skin a*primary colour	Fr-a*	8.4a	−17.9–19.2	6.7a	−2.2–16.0	8.7a	−0.8–16.7	0.649
Fruit skin b*primary colour	Fr-b*	2.6a	−2.1–25.14	4.4a	0.3–14.1	4.8a	−1.08–14.01	0.335
Fruit skin gloss (gloss units)	Fr-Gloss	7.1a	1.9–11.9	10.6b	5.6–17.9	13.7c	8.9–18.5	<0.001
Fruit flesh browning	Fr-Browning	4.0a	1.1–8.4	4.9ab	1.6–9.4	6.3b	0.9–13.2	0.035

a Means separated by different letters within a row are significantly different according to the Student-Newman-Keuls multiple range test at P≤0.05.

Chinese accessions are less vigorous than those of Spain or Sri Lanka, so that the Chinese accessions have lower plant height (P-Height) and smaller leaves (L-Pedicel, L-Length, L-Breadth) than either the Spanish or Sri Lankan eggplants (Table 1). Regarding anthocyanic pigmentation of the vegetative parts of the plant (P-Anthocyans, L-Anthocyans, Fr-CAnthocyans), the highest pigmentation is found in the Chinese eggplants, the lowest in the Sri Lankan ones, while in the Spanish accessions it is intermediate. While the plant prickliness (P-Prickles) has been in general low, Sri Lankan accessions have presented, as a mean, a lower prickliness (P-Prickles) than either the Chinese or Spanish eggplants. Leaves of Sri Lankan accessions had a smaller leaf blade breadth/width ratio (L-Ratio) than the Chinese of Spanish eggplants, and the Chinese accessions had a lower leaf blade lobing (L-Lobing) than the Spanish or Sri Lankan eggplants (Table 1).

For flower traits, Chinese eggplants had, in general, a smaller number of flowers per inflorescence (Fl-Number) than either the Spanish or Sri Lankan eggplants (Table 1). Spanish eggplants had more petals per flower (Fl-Petals) than that of the Chinese or Sri Lankan accessions.

The fruits of Sri Lankan accessions, in general, have higher fruit length (Fr-Length) and lower fruit breadth (Fr-Breadth) and, in consequence, are more elongated (Fr-Ratio) than those of China or Spain (Table 1). Spanish eggplants have a larger part of the fruit covered by the calyx (Fr-CLength) than those of China or Sri Lanka, while the intensity of the fruit skin covered by the calyx (Fr-UnderC) is higher in Sri Lankan accessions, followed by the Spanish ones, and finally by the Chinese accessions,. Contrary to what occurred in a number of Chinese and Spanish accessions, the Sri Lankan eggplants studied did not present chlorophylls in the fruit skin (Fr-Chlorophyll) and therefore presented lower mean values than Chinese or Spanish accessions for this trait (Table 1). Regarding the skin colour, Sri Lankan materials had, on average higher L* (Fr-L*) values (indicating closer to white) than Chinese or Spanish accessions. Fruits of Sri Lankan accessions were, on average, more glossy (Fr-Gloss) than those of the Spanish accessions, which, in turn were also more glossy than the Chinese accessions. Finally, Sri Lankan accessions had a significantly higher flesh browning (Fr-Browning) than Chinese accessions (Table 1).

**Figure 1:**
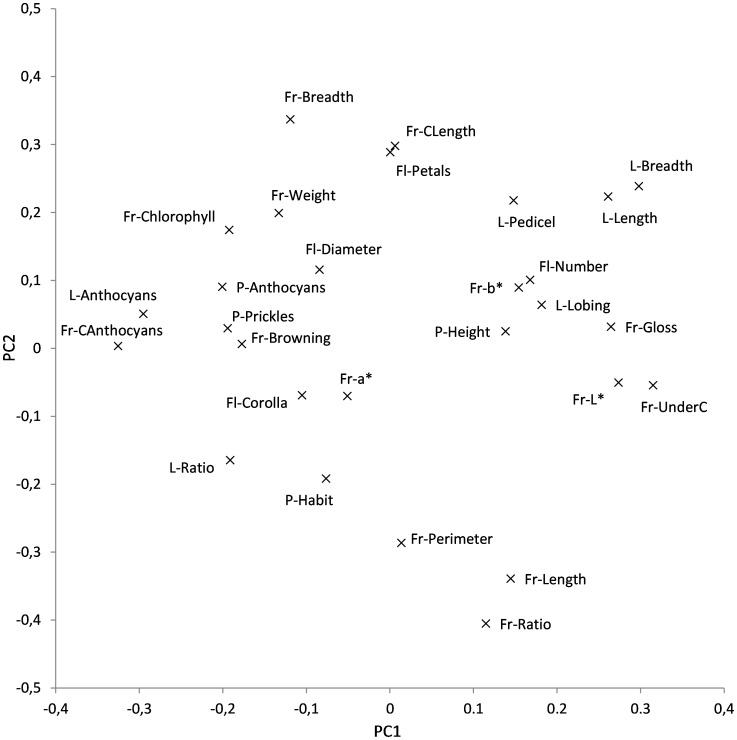
PCA relationships between morphological traits. The two first components (PC1 and PC2) of the principal components analysis account for 22.9% and 16.2% of the total variation, respectively. Results were obtained after the characterization of 52 *S. melongena* accessions from China, Spain, and Sri Lanka using 28 morphological descriptors (see text and Table 2).

According to the scree plot method, a total of five principal components were found to be relevant in the morphological PCA study. These five principal components account for 63.4% of the total variation. However, given that the first and second components account, respectively, for 22.9% and 16.2% of the total variation (Figure 1), and that in the graphical analyses no relevant changes are introduced by including the third, fourth or fifth principal components, we just present and discuss the data referring to the first and second components. The first component was positively correlated to plant vigour traits (L-Breadth, L-Length, L-Pedicel, Fl-Number, and P-Height) as well as to traits related to elongated fruits (Fr-Length, Fr-Ratio, and Fr-Perimeter), and to those associated to the light skin colour, like a high luminosity (Fr-L*), high gloss (Fr-Gloss), and also to a high intensity of the color intensity under calyx (Fr-UnderC) (Figure 1). Negative correlations for this first component were found for traits associated to broad fruits (Fr-Breadth) as well as to traits related to the dark pigmentation (due to content in anthocyanins) of the different plant parts (L-Anthocyanins, Fr-CAnthocyanins, P-Anthocyanins, and Fl-Corolla), or of the fruit caused by the presence of chlorophylls (Fr-Chlorophyll) (Figure 1). Negative correlations of this first component were also found with the leaf breadth/width ratio (L-Ratio), presence of prickles in the plant (P-Prickles), or fruit flesh browning (Fr-Browning). The second component was positively correlated to fruit and flower size (Fr-Weight, Fl-Petals, and Fl-Diameter), to plant vigour traits (L-Breadth, L-Length, L-Pedicel, Fl-Number, and P-Height), to fruit breadth (Fr-Breadth), as well as to the content of chlorophyll in the fruit skin (Fr-Chlorophyll) (Figure 1). This second component was negatively correlated to traits related to elongated fruits (Fr-Ratio, Fr-Length, and Fr-Perimeter) and to prostrate plant habit (P-Habit) (Figure 1).

**Figure 2:**
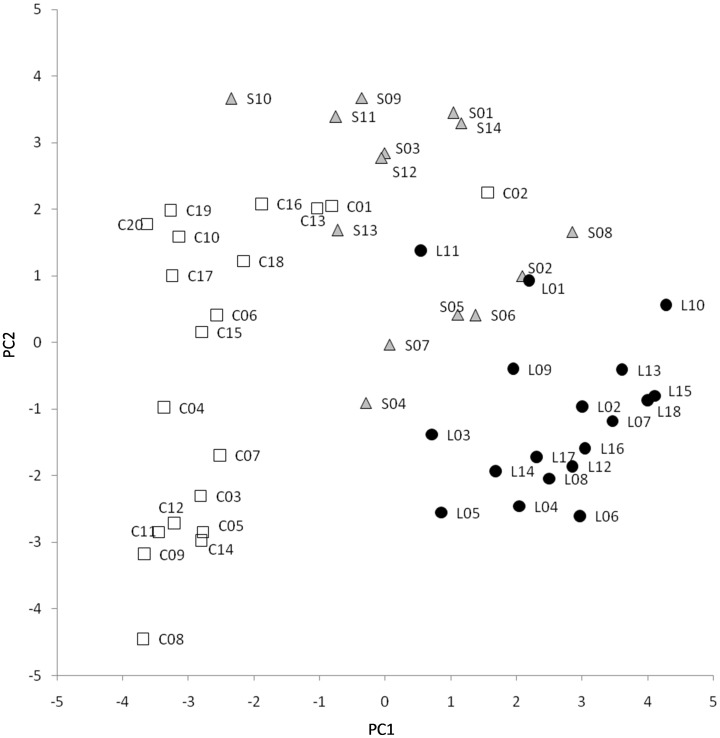
PCA morphology-based relationships between accessions. A total of 52 *S. melongena* accessions from China (white squares), Spain (grey triangles), and Sri Lanka (black circles) (Table 1) were morphologically evaluated with 28 morphological traits (see text and Table 2) and were represented on the two first components (PC1 and PC2) of the principal components analysis (22.9% and 16.2% of the total variation explained by the first and second component, respectively).

The projections of the accessions on the PCA graph show that with the exception of two accessions (C02 and L11) the accessions of China, Spain and Sri Lanka plot in different parts of the PCA graph (Figure 2). The Chinese accessions are, in general, characterized by negative values of the first component, with low values of traits related to plant vigour (L-Breadth, L-Length, L-Pedicel, Fl-Number, and P-Height), dark fruits (low Fr-L* and Fr-Gloss, and high Fr-Chlorophyll), and high pigmentation of the plant (high L-Anthocyanins, Fr-CAnthocyanins, P-Anthocyanins, and Fl-Corolla), as well as for a wide range of values for the second component. Spanish accessions present intermediate-high values for both the first and second components, and therefore are associated to high plant vigour (high L-Breadth, L-Length, L-Pedicel, Fl-Number, and P-Height), large flower and fruit size (high Fl-Petals, Fl-Diameter, Fr-Breadth, Fr-Weight), and negatively to elongated fruits (low Fr-Ratio, Fr-Length, and Fr-Perimeter). Sri Lankan accessions present positive values of the first component and relatively low values of the second component, and therefore are associated to elongated fruits and small flowers and fruits (high Fr-Ratio, Fr-Length, and Fr-Perimeter, and low Fl-Petals, Fl-Diameter, Fr-Breadth, and Fr-Weight) with light skin fruit (high Fr-L* and Fr-Gloss and low Fr-Chlorophyll) and low plant pigmentation (L-Anthocyanins, Fr-CAnthocyanins, P-Anthocyanins, and Fl-Corolla) (Figure 2). The outlier Chinese accession C02 is, together with C01, the only accession from this origin that does not have anthocyanins in the fruit (high Fr-L* and Fr-UnderC values), and is also characterized by large leaves (high L-Pedicel, L-Length, L-Breadth) and high fruit flesh browning (Fr-Browning), which results in high values of the first component. Also, the outlier Sri Lankan accession L11 is the only one in this group that does not present elongated fruits (high L-Breadth and low Fr-Length and Fr-Ratio) and therefore has high values for the second component of the PCA and is intermingled with the Spanish accessions (Figure 2).

The discriminant analysis of the 28 morphological traits revealed that 100% of the accessions were correctly classified to their country of origin. The forward stepwise discriminant analysis showed that a correct classification of all the accessions could be achieved using a minimum set of six traits: L-Length, Fr-L*, Fr-UnderC, Fr-b*, P-Prickles, and Fr-Weight. The two discriminating functions for this model were highly significant (P<0.001). For all these traits, with the exception of Fr-b*, important and highly significant (P<0.001) differences were found among origins (Table 1).

### SSR Characterization

The twelve SSR loci evaluated were polymorphic in the materials evaluated and amplified 110 alleles (average of 9.2 alleles per locus) (Table 2). The number of alleles per locus ranged between five for CSM54 and CSM73 and 16 for CSM30. PIC values ranged between 0.251 for CSM43 and 0.722 for CSM30, with an average value of 0.574. A unique genetic fingerprint was obtained for each of the individual accessions.

**Table 2:** SSR markers, number of alleles per locus for all the samples and for each origin (China, Spain, and Sri Lanka), number of private alleles (i.e., present in one or more accessions of each origin), and PIC value for each SSR locus.

SSR locus	All samples	China		Spain		Sri Lanka		PIC
	Alleles (n)	Alleles (n)	Private alleles (n)	Alleles (n)	Private alleles (n)	Alleles (n)	Private alleles (n)	
CSM7	6	4	1	3	0	4	1	0.489
CSM27	11	4	1	4	2	8	4	0.569
CSM30	16	9	4	7	3	6	4	0.722
CSM31	12	7	4	4	2	6	3	0.626
CSM32	12	5	2	6	2	6	4	0.598
CSM36	6	3	1	2	0	5	3	0.351
CSM43	6	1	0	3	2	4	3	0.251
CSM44	11	3	1	5	2	7	5	0.606
CSM52	6	3	0	4	1	4	1	0.550
CSM54	5	5	3	2	0	2	0	0.422
CSM73	5	1	0	4	2	4	1	0.331
CSM78	14	7	3	6	2	7	5	0.720
*Mean*	*9.2*	*4.4*	*1.7*	*4.2*	*1.5*	*5.25*	*2.8*	*0.574*
Total	110	52	20	50	18	63	34	---

**Figure 3:**
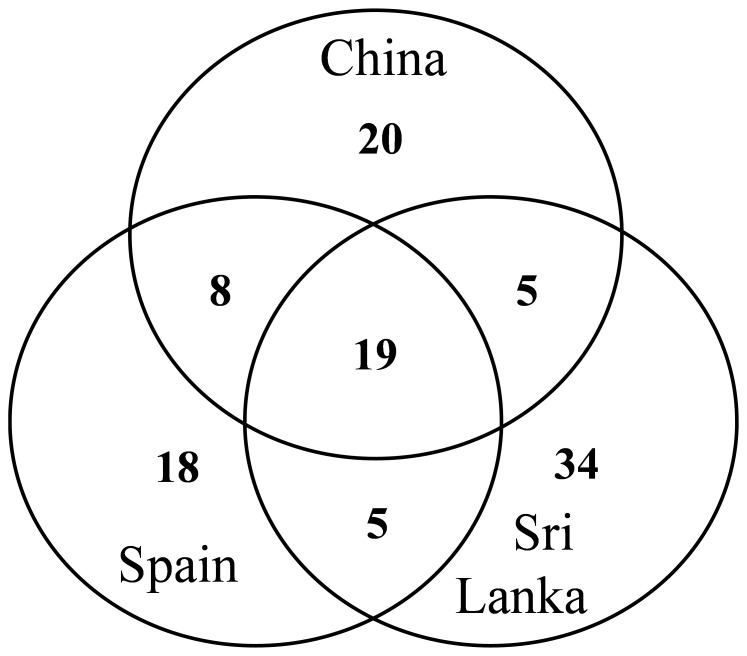
SSR alleles in the different origins. The figures indicate the number of SSR alleles private to each of the secondary centers of diversity, as well as the number of alleles shared by accessions from two or the three different origins.

When considering the number of alleles present in accessions from different origins, the Sri Lanka materials presented the highest number of alleles (63), followed by Chinese (52), and Spanish (50) accessions, which corresponds, respectively, to 57.3%, 47.3%, and 45.5% of the total number of alleles detected (Table 2). However, no private and universal SSR alleles were found for any of the three origins. For the materials of Sri Lanka, a total of 34 alleles were private (i.e., unique to one or more accessions of this group), while for China and Spain the number of private alleles was of 20 and 18, respectively (Table 2). Most of these private alleles specific to each origin were present in low frequencies, so that when considering each of the origins separately the average value of the frequency of the private alleles was of 0.077, with a maximum frequency value of 0.308. Eighteen alleles were present in all the groups, while 9 were exclusive of China and Spain, 5 of China and Sri Lanka, and 5 to Spain and Sri Lanka (Figure 3).

**Figure 4:**
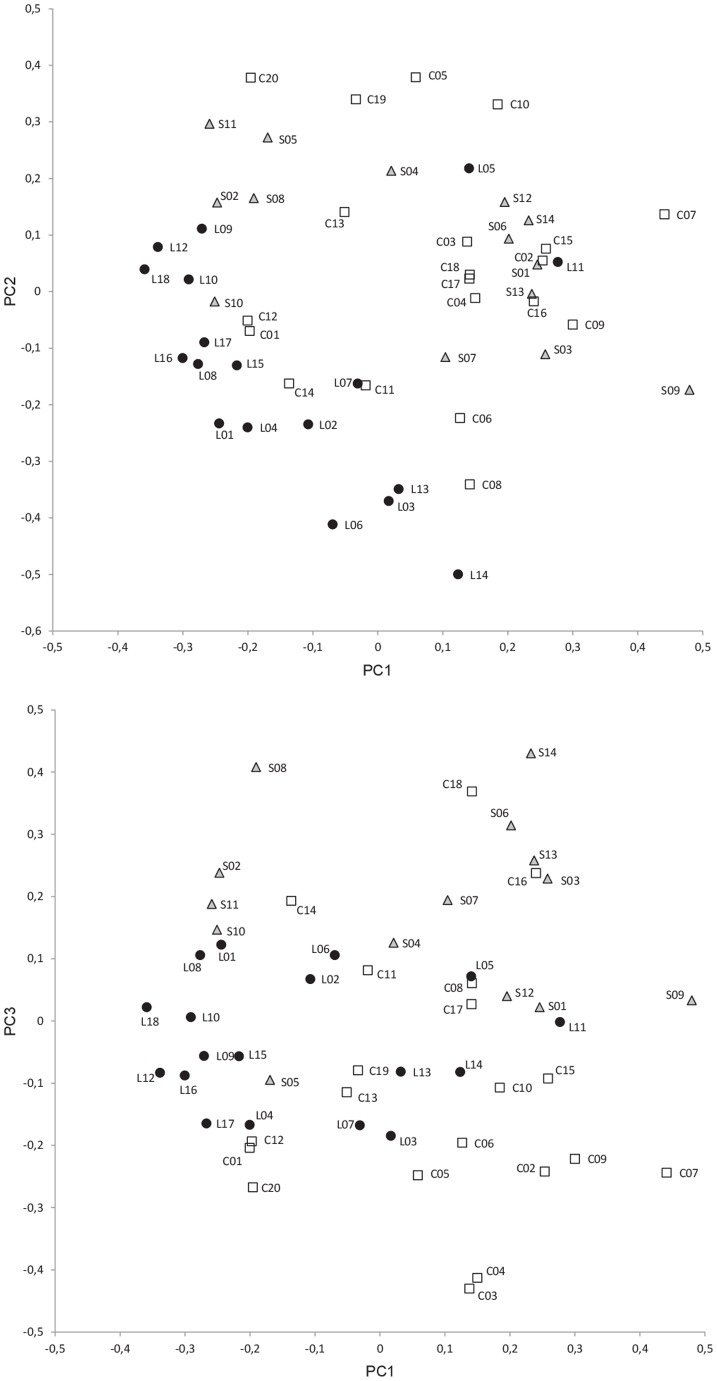
PCA SSR-based relationships between accessions. A total of 52 *S. melongena* accessions from China (white squares), Spain (grey triangles), and Sri Lanka (black circles) (see Table 1) were evaluated using 12 polymorphic SSRs (see text and Tables 3 and 5) and were represented on the three first components (PC1, PC2 and PC3) of the principal components analysis (22.0%, 19.1%, and 17.9% of the total variation explained by the first, second, and third principal components, respectively). Scatterplots show the projections of the accessions on the first and second principal components (above) and on the first and third principal components (below).

The first and second components of the PCA analysis performed with SSR data account for 22.0% and 19.1% of the total variation, respectively. Although in this analysis accessions of the different origins are intermingled (Figure 4), accessions from China and Sri Lanka are mostly distributed in different areas of the plot. With the exception of L05 and L11, accessions from Sri Lanka have a combination of low and moderate values for the first and second components; on the other hand, with the exception of C01, C11, C12, and C14, accessions from China present positive values of either the first or second components (Figure 4). Accessions from Spain are scattered all over the graph with the exception of the bottom part of the plot (i.e., the area with values <−0.2 for the second component). Inclusion of a third component in the analysis (17.9% of the total variation explained by this component) shows that no accessions from Spain present low values for the third component (all accessions have values >−0.15), while no accessions from Sri Lanka present high values for this component (all accessions have values <0.15). On the other hand, accessions for China present a wide range of values for the third component (Figure 4).

**Figure 5:**
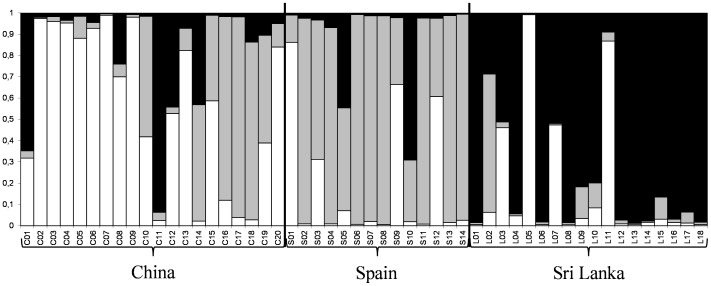
Assignment tests of accessions to populations. The estimated number of populations (parameter *K*) was set to 3 in the STRUCTURE software (Pritchard et al., 2000). Accessions are organized according to their origin (China, Spain, and Sri Lanka). The three populations are named according to the origin of the majority of the accessions constituting each population (CH for China, SP for Spain, and SL for Sri Lanka). Each accession is represented by a horizontal line, which is partitioned into colored segments (white for the CH population, grey for the SP population, and black for the SL population).

The Evanno’s test indicated that the most informative number of populations (*K*) was 3. The inferred population structure for *K* = 3 obtained with the STRUCTURE software showed that most of the accessions of each origin are assumed to belong to the same population (Figure 5). Therefore, we have named these populations as CH, SP, and SL, corresponding to populations containing mostly Chinese, Spanish, and Sri Lankan accessions, respectively. In this respect, when the highest value for the membership coefficient (*q_i_*) is used to assign one accession to a population, for the Chinese accessions, 14 out of the 20 accessions are assigned to the CH population; for the rest of Chinese accessions, C14, C16, C17, and C18 are assigned to the SP population, and C01 and C11 to the SL population. In the case of the Spanish accessions, 10 out of the 14 accessions are assigned to the SP population; three of the rest of Spanish accessions (S01, S09, and S12) to the CH population, while the remaining one (S10) to the SL population. Finally, the 15 out of the 18 Sri Lankan accessions are assigned to the SL population; two out of three of the rest of accessions (L05 and L11) to the CH population, and the last one (L02) to the SP population (Figure 5). For most of the accessions (69.2%), the membership coefficient *q_i_* to one of the populations was higher than 0.8, while the rest (30.8%) could be considered as admixed (*q_i_*≤0.8). Seven of the admixed accessions were from China (C01, C08, C12, C10, C14, C15, and C19), five from Spain (S03, S05, S09, S10, and S12), and four from Sri Lanka (L02, L03, L07, and L10) (Figure 5).

The three origins present a high level of total genetic diversity (H_T_), which ranges from H_T_ = 0.4943 for Chinese accessions to H_T_ = 0.5400 for Sri Lankan accessions (÷). The partition of the total diversity shows that most of the genetic diversity is found within each of the groups (D_ST_ = 0.0481 and H_S_ = 0.4909), which results in a moderate value of the relative magnitude of genetic differentiation (G_ST_ = 0.0892). However, the standardized G_ST_ (G’_ST_) reached a much higher value (G’_ST_ = 0.2657) (Table 3). The values of heterozigosity for individual accessions were low, with an average value of 0.052, with non-significant differences among origins (P>0.05).

The lowest genetic distance (and consequently highest genetic identity) values are found between the Chinese and Sri Lankan origins, with a value of 0.1204 (Table 4). The genetic distance values of Spanish and Chinese origins and of Spanish and Sri Lankan eggplants are similar, with values of 0.1613 and 0.1604, respectively (Table 4). Correlations obtained from the Mantel test between the morphological and SSR distances were low (r = 0.044) and non-significant (t = 0.5729; P = 0.7166).

## Discussion

Morphological and molecular data are not only complementary for the study of the diversity and relationships of eggplants from these distant centers of diversity, but also synergistic, as information of interest becomes conspicuous when comparing both type of data. Several studies are available on the relationships between morphological and molecular data in eggplant, but these studies have been restricted to specific cultivar groups [4], [28]–[29], to study Spanish accessions [19], or to compare both type of data for predicting the performance of hybrids [5]. In this respect, our study is the first one to study simultaneously the morphological and molecular diversity of a significant number of eggplant accessions from geographically distant centers of diversity of eggplant.

**Table 3:** Total genetic diversity (H_T_), among groups genetic diversity (D_ST_), within groups genetic diversity (H_S_), relative magnitude of genetic differentiation (G_ST_) and standarized G_ST_ (G’_ST_), estimated from SSR data for the eggplant accessions according to their origin.

Group	Samplesize	H_T_	D_ST_	H_S_	G_ST_	G’_ST_
Total	52	0.5390	0.0481	0.4909	0.0892	0.2697
Origins						
China	20	0.4943				
Spain	14	0.5286				
Sri Lanka	18	0.5400				

### Morphological Diversity

The morphological data show that within each of the origins, a considerable diversity exists, confirming what had already been observed within Spanish eggplants [19]. For most morphological traits significant differences among origins are found for most traits, and eggplants from the different origins mostly plot in different areas of the PCA graph, showing that accessions from each of the three secondary centers of diversity are morphologically differentiated and present a typical combination of traits [28], [29]. In this way, a combination of just six morphological traits is enough to correctly assign any of the accessions to its origin. Amazingly, in this study we have found that the number of differences between eggplants from China and Sri Lanka, has been much greater than those between any of the former origins and Spanish eggplants. These results indicate that although cultivated eggplants have often been classified into “Western” or “Occidental” and “Asian” or “Oriental” cultivar groups [3], [6], [10], [35], the real situation, in particular of the “Asian” eggplants may be more complex, with several groups with important morphological differences. For example, in general, the Chinese accessions used in our study have more anthocyanin pigmentation, are more prickly, have darker fruits, with more chlorophyll in the skin, and less vigorous, with smaller leaves, and less elongated and glossy fruits than Sri Lankan accessions. The few outlier accessions that in the morphological PCA group with accessions from other origins (C02 and L11) present a few morphological characteristics that differ greatly from the rest of accessions of their origin (e.g. lack of anthocyanin pigmentation, large leaves, and high fruit flesh browning in C02, and round fruits in L11). However, for the rest of traits present values similar to those of the Chinese (C02) and Sri Lankan (L11) accessions and therefore, when using the discriminant traits are correctly classified to their origins.

**Table 4:** SSR-based estimates of genetic distances (above the diagonal) and genetic identities (below the diagonal) between different eggplant origins.

Origin	China	Spain	Sri Lanka
China		0.1613	0.1204
Spain	0.8510		0.1604
Sri Lanka	0.8813	0.8514	

The wide range found for most morphological traits within each of the origins suggests that genetic drift may not have been an important force in the morphological differentiation of eggplant within each of the three secondary centers of diversity [40]. On the other hand, the morphological differentiation found among origins may indicate that natural selection and/or, more likely, artificial selection may have been responsible for the combination of morphological differences characteristic of accessions from different secondary centers of diversity [41]. Some of the traits for which significant differences exists among origins (e.g., prickliness, anthocyanin pigmentation, fruit chlorophyll, presence of pigmentation under the calyx) are monogenic or oligogenic and present a high degree of penetration and expression [3], [42]; therefore, the frequencies of phenotypes change quickly in response to selection. In consequence, the fact that for these traits the means are different, even though a wide range of variation is present in each origin, suggests that artificial selection, very likely linked to different uses or to different management practices, may have been responsible for the morphological differentiation among origins. This suggests that ethnobotanical aspects may have had an important role in the differentiation of eggplant landraces from separate regions.

The important differences among origins for morphological traits may be relevant for eggplant breeding, and indicates that sources of variation of interest for several relevant agronomic traits of materials from a center of diversity may be found in exotic materials from other centers of diversity. For example, when considering some traits of interest for breeding modern cultivars [3], in general Chinese accessions seem to be a good source of variation for large fruits and low fruit flesh browning, Spanish ones for vigorous (e.g., high with large leaves) plants, and Sri Lankan accessions for low prickliness and high glossiness.

### Molecular Diversity

The high morphological variation in the materials studied is matched by high levels of molecular diversity. In this sense, we have found that the 12 genomic SSR loci were polymorphic and allowed the detection of a total of 110 SSR alleles (9.2 alleles/polymorphic locus). These 12 SSR loci were developed by Vilanova et al. [35] and were selected for the present study for its high polymorphism. However, the level of polymorphism found here has been higher than that found in the original work of Vilanova et al. [35], in which a total of 92 alleles (7.7 alleles/polymorphic locus) were detected in 22 eggplant materials from different cultivar types and origins. In other studies, the level of SSR polymorphism has been much lower. For example, Muñoz-Falcón et al. [38] in a study of 42 eggplant accessions from different origins and cultivar groups used 49 SSR loci (17 corresponding to genomic SSRs and 32 to EST-SSRs) of which only 21 were polymorphic and which allowed the detection of 85 alleles (4.1 alleles/polymorphic locus). In a similar study, Muñoz-Falcón et al. [4] found polymorphism for 11 out of 14 SSR loci (2 genomic SSRs and 12 EST-SSRs), giving a total of 61 alleles (4.7 alleles/polymorphic locus) in a collection of 44 eggplants. Stàgel et al. [32] found that only 11 out of 38 EST-SSR loci were polymorphic in a collection of 38 eggplant cultivars of different origins and allowed the detection of 39 alleles (3.5 alleles/polymorphic locus). Ge et al. [23] in a study of 88 EST-SSRs of 42 Asian accessions found polymorphism for 79 of them, with a total of 323 polymorphic alleles (4.1 alleles/polymorphic locus). Also, when we consider each of the three origins, a high diversity is found, and the number of alleles per polymorphic locus is of 4.3, 4.2, and 5.3 for accessions from China, Spain, and Sri Lanka, respectively, which is a confirmation that these three countries can be considered as secondary centers of diversity [19]–[21]. The fact that a high number of private alleles are found in each of the origins we have studied, although most of them are present at low frequencies, has important implications for the conservation of the genetic diversity. For example, it suggests that the establishment of core collections for this crop should include accessions from different origins so that most of the allelic diversity is represented [43].

The multivariate analysis of SSR data shows a less clear picture of the differentiation among centers of diversity than the analysis of morphological data, with a higher degree of admixture among accessions of different origins in the SSR PCA plots than in the morphological PCA graph. In any case, the combination of the first and second components separate most of the Sri Lankan accessions from most of the Chinese accessions, while the third component differentiates mostly the Spanish and Sri Lankan accessions. Therefore, despite the fact that some of the accessions are intermingled, differentiation is observed among the three origins in the PCA analysis. The Bayesian-based analysis without a priori assignment of accessions to populations resulted in the creation of three populations, each of which was formed mostly by accessions from a single origin, which suggests an important differentiation among origins [25], [26]. The results of the PCA analysis show a good agreement with the population structure analysis. In this respect, accessions from one origin that in the PCA analysis plotted closer to most of the accessions of another origin, were assigned to the population formed mostly by accessions of this latter origin in the population structure analysis. More than two thirds of the accessions were clearly assigned to one of the populations, as they presented a membership coefficient *q_i_* to one of the populations higher than 0.8 [25], [44]; the rest of accessions could be considered as admixed, although in most cases were basically an admixture of only two populations. Accessions from one origin (e.g., China) assigned in the population structure analysis to populations mostly composed by accessions from another origin (e.g. Spain or Sri Lanka) were mostly not admixed accessions, which may indicate that introduction of these materials have taken place in relatively recent times. Also, the fact that eggplant is fundamentally autogamous [45] may have helped to maintain the genetic integrity of the materials introduced from another center of diversity.

The analysis of the genetic diversity with the total diversity (H_T_) parameter [46] shows that the total diversity present in each of the origins is high and similar to the total diversity present in the whole collection, which is an indication of the high diversity present in each origin. This result is somewhat surprising, as the diversity present in a small country, like Sri Lanka, is even higher than the diversity found in a large country, like China. Given that Sri Lanka is close to India, which is part of the primary center of diversity [3], [9], [10], may have allowed the accumulation of a high diversity in Sri Lanka.

Despite the fact that the genetic diversity among groups (D_ST_) is considerably lower than the genetic diversity within groups (H_S_), the standardized genetic differentiation (G’_ST_), which is more adequate than G_ST_ for SSR data [47] is greater than 0.25, which is a moderate value and shows that there is a considerable differentiation among the three origins [25], [47], [48]. When considering the genetic relationships among the three origins, the lowest genetic distance is found between Chinese and Sri Lankan accessions, while the genetic distance between Spanish and Chinese or Sri Lankan accessions are similar. Given that China and Sri Lanka are geographically closer than to Spain probably has contributed to a greater exchange of materials and genetic flux among these two countries, resulting in lower genetic distances.

The heterozygosity values observed were low, and similar to the values found by others in eggplant landraces of the black type [4]. This result was expected, given the high degree of autogamy of eggplant 3,45, which results in the fixation of alleles in homozygosis in populations in which there is no artificial control of pollination.

### Comparison of Morphological and Molecular Diversity

The correlations between morphological and SSR molecular data have not been significant, which is contrast to the moderate value (r = 0.38) found by Muñoz-Falcón et al. [4] when comparing morphological and SSR data in a study of black eggplants which included modern F1 hybrids, old non hybrid cultivars, Spanish landraces, and Non-Spanish landraces. Significant correlations between morphological data and AFLPs were also found in the former study, and also by Prohens et al. [19] in a study of the diversity of Spanish eggplants. The correlations between morphological and molecular data are usually very variable and dependent, among others, on the plant material, morphological descriptors, and molecular markers used. In this respect, in other solanaceous crops, like pepper [49] or potato [50] no significant correlations were found among both types of data.

The lack of correlations in our collection suggests that both types of data sample different levels of diversity and, therefore, both of them should be considered for the management and conservation of germplasm [51]. In this respect, morphological markers in eggplant sample traits for which the variation is usually controlled by a few genes or QTLs [3], [42], while genomic SSRs detect differences in the length of short tandem repeated sequences mostly situated in non-coding regions of the genome [36], [37]. Wendel and Doyle [52] suggested that the lack of correlation between morphological and molecular data may be caused by the fact that both types of markers follow different evolutionary paths.

### Conclusions

The results show that eggplant accessions from the three geographically distant secondary centers of diversity studied (China, Spain, and Sri Lanka) present a wide morphological and molecular diversity. At the morphological level, a clear differentiation exists among the different origins, indicating that different selection criteria have been applied in each secondary center of diversity, leading to a typical syndrome of traits for each origin. The molecular data also show that considerable differentiation exists at the molecular level among the three origins, although in this case there is evidence of migration between the different origins, as revealed by the study of the population structure. The lack of correlation between morphological and molecular diversity shows that both types of data provide complementary information and that both of them should be taken into account in the management of germplasm and formation on core collections [43], [51]. The results also have importance for eggplant breeding, as it shows that sources of variation of interest can be found in the materials evaluated, and also suggests that crossing among individuals assigned to different populations may result in heterotic hybrids [5]. Finally, these results are also important for understanding the evolution and domestication of eggplant [9], [10].

## Materials and Methods

### Permissions

No specific permits were required for the described field studies, which took place in an experimental field plot at the Universitat Politècnica de Valencia. This field plot is used by the authors of this paper affiliated to the aforementioned institution (MH, SV, MP, PG, and JP) for field trials for characterization of germplasm of cultivated species.

### Plant Materials

Fifty-two accessions of *S. melongena*, of which 20 originated in China (C codes), 14 in Spain (S codes), and 18 in Sri Lanka (L codes) were used for the present study (Table 5). The materials correspond to local landraces or to selections within the local landraces and were chosen trying to represent the diversity of local materials of *S. melongena* of each country. The criteria used for choosing the accessions was based on previous available morphological and genetic data (for the Spanish accessions) [4], [19], [28], [35], on distribution across geographic range, and also on availability of seeds. The plant material used is part of the collection of the Instituto de Conservación y Mejora de la Agrodiversidad Valenciana (Valencia, Spain).

**Table 5:** Plant materials used for the study of morphological and molecular (SSR) variation of a collection of eggplants from China, Spain, and Sri Lanka.

Accession name	Code	Place of origin
*China*		
Qi Xian Hei You Guan	C01	Qi
Tuo Cheng Qing Qie	C02	Tuocheng
Zao Xian Qie	C03	Acheng
Qie Zie	C04	Aihui
Zi Chang Qie	C05	Hubei
Tuan Qie	C06	Zhuix
Wu Xian Qie	C07	Anxiang
He Xian Qie	C08	Chenzhou
Yong Ji Xian Qie	C09	Yongji
Zi Yuan Qie	C10	Taoan
Niu Jiao Qie	C11	Changzhou
Niu Jiao Qie	C12	Jiangyin
Hong He Bao Qie	C13	Yichun
Yu Jiang Chang Xian Qie	C14	Yujiang
Bao Tou Niu Xin Qie	C15	Baotou
Tian Jin Da Min Qie	C16	Yinchuan
Tian Jin Er Min Qie	C17	Yinchuan
Cao Xian Yuan Zie Qie	C18	Cao
De Zhou Duan Ba Hong Qie	C19	Dehou
ASI-S-1	C20	Beijing
*Spain*		
AN-S-24	S01	Jaén
V-S-10	S02	Alicante
AN-S-3	S03	Córdoba
MU-S-7	S04	Murcia
B-S-3	S05	Baleares
MU-S-4	S06	Murcia
V-S-3	S07	Valencia
V-S-2	S08	Gandía
V-S-9	S09	Alicante
MU-S-8	S10	Murcia
MU-S-6	S11	Murcia
NC056471	S12	Murcia
H15	S13	Almagro
AN-S-23	S14	Jaén
*Sri Lanka*		
BW11	L01	Bombuwela
SM 164	L02	Gannoruwa
Thinnevelly Purple	L03	Jaffna
8890	L04	Matara
SA7MTE2	L05	Unknown
5124	L06	Gampaha
7517	L07	Colombo
1624	L08	Galle
558	L09	Nuwara Eliya
799	L10	Matale
8891	L11	Matara
1139	L12	Puttlam
2287	L13	Unknown
Ridiyagama	L14	Hambantota
Farmer Lenairi	L15	Anuradhapura
Kaluthavelly	L16	Batticaloa
Ampara	L17	Ampara
Welimada	L18	Welimada

For each accession five plants raised from seed were grown in an open-air field plot (39°28’55” N; 0°20’11” W) in Valencia (Spain) following a completely randomized experimental design. Plants were spaced 1 m between the rows and 0.8 m apart in the row. The standard horticultural practices for eggplant production in the area of Valencia were followed.

### Morphological Characterization

Individual plants were characterized using 23 primary descriptors, most of which were developed by EGGNET [4], [19], [30]. These descriptors include plant (P), leaf (L), flower (Fl), and fruit (Fr) characteristics. Thirteen traits corresponding to these primary descriptors are quantitative: plant height (cm; P-Height), leaf pedicel length (cm; L-Pedicel), leaf blade length (cm; L-Length), leaf blade breadth (cm; L-Breadth), leaf blade length/breadth ratio (L-Ratio), flowers per inflorescence (Fl-Number), flower diameter (cm; Fl-Diameter), petals per flower (Fl-Petals), fruit longitudinal section perimeter (cm; Fr-Perimeter), fruit length (cm, Fr-Length), fruit breadth (Fr-Breadth), fruit length/breadth ratio (Fr-Ratio), fruit weight (g; F-Weight). The other ten traits are measured in a scale with predetermined values (Table 6), and correspond to EGGNET descriptors [4], [19], [30]. Apart from these primary descriptors, the skin colour and brightness, as well as the fruit flesh browning were also measured objectively in at least three fruits per plant. For the skin colour the L* (Fr-L*), a* (Fr-a*), and b* (Fr-b*) Hunter colour coordinates of the predominant (primary) colour of the skin were assessed with a Minolta CR300 (Minolta Co., Osaka, Japan) chromameter. The skin gloss (gloss units; Fr-Gloss) was measured with a Novo-Curve Elcometer 400 (Elcometer, Manchester, UK) glossmeter. Finally, for the fruit flesh browning (Fr-Browning), the flesh colour in the central part of a transversal section of the fruit was measured with the CR300 chromameter at 0 min and 10 min after being cut, and the flesh browning was measured as the difference between the Hunter colour coordinate L* (luminosity) at 0 and 10 min [53], so that the higher the value of the difference the greater the browning.

**Table 6:** Morphological traits measured in a scale with pre-determined values of the descriptor states, and description of the scale used for the study of morphological variation in the eggplant accessions studied [4], [19], [30].

Traits	Codes	Range (scale)
Plant growth habit	P-Habit	1–9 (1 = upright; 9 = prostrate)
Shoot tip anthocyanins intensity	P-Anthocyans	0–9 (0 = absent; 9 = very strong)
Stem prickles	P-Prickles	0–9 (0 = absent; 9 = very many)
Leaf blade lobing	L-Lobing	1–9 (1 = very weak; 9 = very strong)
Leaf anthocyanins intensity	L-Anthocyans	0–9 (0 = absent; 9 = very strong)
Corolla colour	Fl-Colour	0–7 (0 = White; 7 = blue)
Relative fruit calyx length	Fr-CLength	1–9 (1 = very short (<10%); 9 = very long (>10%))
Fruit calyx anthocyanins intensity	Fr-CAnthocyans	0–9 (0 = absent; 9 = very strong)
Fruit color intensity under calyx	Fr-UnderC	0–9 (0 = none; 9 = very strong)
Fruit skin chlorophyll	Fr-Clorophyll	0–9 (0 = none; 9 = very strong intensity)

### Molecular Characterization

Genomic DNA was extracted from a mixture of young leaf from the five plants morphologically evaluated according to the CTAB method procedure [54]. The quality of DNA was checked on 1% agarose gels and the DNA concentrations estimated using a Nanodrop ND-1000 (Nanodrop Technologies, Wilmington, Delaware, USA) spectrophotometer.

For the present study we used twelve simple sequence repeat (SSR) markers (Table 7) that proved to be highly polymorphic and that presented high PIC values in eggplant [35]. The map position of nine out of these 12 SSRs is known (Table 7). SSRs were amplified following the M13-tail method described by Schuelke [55] to facilitate the incorporation of a dye label during PCR. Amplifications were performed in a total volume of 12 µl, with 10 ng DNA, 1 mM MgCl_2_, 0.05 µM of forward primer, 0.25 µM of reverse primer, 0.2 µM fluorescent-labelled M-13 primer, 0.2 mM dNTPs and 1 unit of *Taq* polymerase in 1X PCR buffer. Conditions of the PCR amplification were as follows: 1 cycle for 2 minutes at 94°C, 35 cycles of 15 s at 94°C, 30 s at appropriated annealing temperature (Table 7), 45 s at 72°C, followed by 10 min extension at 72°C. SSR alleles were resolved on an ABI PRISM 3100 DNA (Applied Biosystems, Carlsbad, California, USA) genetic analyzer using GeneScan 3.7 (Applied Biosystems) software and precisely sized using GeneScan 500 LIZ molecular size standards with Genotyper 3.7 (Applied Biosystems) software.

**Table 7:** Primer sequences, expected size, annealing temperature, linkage group, and map position [35], [39] of the twelve SSR markers used for molecular characterization of the materials studied.

SSR locus	Repeat	Primer sequence (5′-3′)	Annealing temperature	Linkage group
CSM7	CT	F- CGACGATCACCTTGATAACG	58.6	Unknown
		R- CCTAAATGCAGAGTTTCCAAAG		
CSM27	GA	F- TGTTTGGAGGTGAGGGAAAG	60.0	3^a^
		R- TCCAACTCACCGGAAAAATC		
CSM30	CT	F- CACTGTTCCTGGTTGCTGTG	60.1	9^b^
		R- TTTAGCTTTAGCCCATCTACCG		
CSM31	AG	F- CAACCGATATGCTCAGATGC	59.8	1^c^
		R- GCCCTATGGTCATGTTTTGC		
CSM32	AG	F- TCGAAAGTACAGCGGAGAAAG	59.6	4
		R- GGGGGTTTGATTTTCATTTTC		
CSM36	GA	F- CCTCAATGGCAGTAGGTCAGA	60.1	9^b^
		R- GTTCTTTGAGCCTCCAGTGC		
CSM43	AG	F- ATTTTAACCCCGGGAAAATG	59.6	1^c^
		R- ACCGCTTCTAGGTTTTGCAC		
CSM44	AG	F- CGTCGTTGTAACCCATCATC	58.7	3^a^
		R- TTGCCAAATTCCTTGTGTTC		
CSM52	TC	F- CTTGGGTCACAAAAGGTTCC	59.7	Unknown
		R- TCACCGAAAAAGATCCAACC		
CSM54	GA	F- ATGTGCCTCCATTCTGCAAG	61.1	9^b^
		R- TGGGTGGGATGCTGAGTAAG		
CSM73	CT	F- TTCAACATAGCCTGGACCATT	60.0	Unknown
		R- AATGCAGGGTTTGGACTTCA		
CSM78	CT	F- AGGGAGGAGCTCTCGTGTG	60.2	10
		R- CAATAACGTAGCTTAATTACTCCCAAG		

a Markers CSM27 and CSM44 are positioned at 10.8 cM and 88.7 cM, respectively, in linkage group 3.

b Markers CSM30, CSM36 and CSM54 are positioned at 29.3 cM, 71.2 cM, and 0.0 cM, respectively, in linkage group 9.

c Markers CSM31 and CSM43 are positioned at 182.0 cM and 161.8 cM, respectively, in linkage group 1.

### Data Analysis

The range and mean values for the morphological traits for each of the groups of Chinese, Spanish, and Sri Lankan eggplants were calculated from the means of each accession. Analyses of variance were used to detect differences for the traits studied among the three groups of accessions. Kolmogorov-Smirnov and Bartlett tests were performed to test, respectively, the normality of data and homogeneity of variances among different origins. Principal components analysis (PCA) were performed for standardised morphological and agronomic data using pairwise Euclidean distances among accessions. Eigenvalues were calculated for each of the principal components, and relevant components were identified using the spree plot method [56]. Discriminant analysis was used to study the percentage of cases correctly classified, and the forward stepwise procedure was used for selecting the minimum subset of variables for discriminating among the three groups.

For the SSR data, pairwise genetic similarities were estimated using the Dice (Sorensen) similarity coefficient. Principal components analyses (PCA) were performed using the pairwise genetic similarities. Possible population structure associated to origin and likelihood of assignment of each accession to population was estimated using the Bayesian-based model procedure implemented in the software STRUCTURE v2.3.3 [57]. The analysis was carried out using a burning period of 10000 iterations. We tested a continuous series of *K*, from 1 to 10, in 10 independent runs [58]. No prior knowledge about the population of origin was introduced. The most informative *K* was identified using the statistic Δ*K* [59]. Subsequently, population structure was inferred for *K* = 3 and using 50000 iterations. Genetic diversity was estimated with the total diversity (H_T_) [46]. Total diversity was partitioned into diversity among origins (D_ST_), and within origins (H_S_). The relative magnitude of genetic differentiation among origins (G_ST_) was calculated as the ratio D_ST_/H_T_ [46]. The standarized G_ST_ (G’_ST_), which standardizes the observed G_ST_ value with the maximum possible value that G_ST_ could obtain given the amount of observed diversity, was calculated as (G_ST_(1+H_T_))/(1−H_S_) [47]. For each accession, heterozygosity values for SSRs were calculated as 1−∑(p_i_^2^), where p_i_ is the frequency of the ith allele [60]. Genetic distances and identities among groups were calculated according to Nei [61]. Correlations between morphological and SSR distance matrices were investigated using a Mantel [62] test.
